# The Effect of Very High versus Very Low Sustained Loading on the Lower Back and Knees in Middle Life

**DOI:** 10.1155/2013/921830

**Published:** 2013-09-05

**Authors:** Yael Milgrom, Charles Milgrom, Naama Constantini, Yaakov Applbaum, Denitsa Radeva-Petrova, Aharon S. Finestone

**Affiliations:** ^1^Department of Orthopaedics, The Hadassah University Hospital, Hebrew University Medical School, 91129 Jerusalem, Israel; ^2^The Sackler School of Medicine, Tel Aviv University, 69978 Tel Aviv, Israel

## Abstract

To evaluate the effect of the extremes of long term high and low physical activities on musculoskeletal heath in middle age, a historical cohort study was performed. The MRI knee and back findings of 25 randomly selected subjects who were inducted into the armed forces in 1983 and served at least 3 years as elite infantry soldiers were compared 25 years later, with 20 randomly selected subjects who were deferred from army service for full time religious studies at the same time. Both cohorts were from the same common genome. The two primary outcome measures were degenerative lumbar disc disease evaluated by the Pfirrmann score and degenerative knee changes evaluated by the WORMS score. At the 25-year follow up, the mean Pfirrmann score (8.6) for the L1 to S1 level of the elite infantry group was significantly higher than that of the sedentary group (6.7), (*P* = 0.003). There was no statistically significant difference between the WORMS knee scores between the two cohorts (*P* = 0.7). In spite of the much greater musculoskeletal loading history of the elite infantry cohort, only their lumbar spines but not their knees showed increased degenerative changes at middle age by MRI criteria.

## 1. Introduction

Degenerative changes in the lower back [1] and knees [2] are frequently present in middle age. The cause of these degenerate changes is multifactorial. It is based on genetics and influenced by lifestyle and injuries [3, 4]. To maximize musculoskeletal health regular exercise [5], proper diet [6] and maintenance of an appropriate body weight are recommended [7]. The long term effect of sustained extremes of exercise, either very high or very low loading, on the health of knees and the lower back is not fully defined.

Elite infantry soldiers undergo long and demanding physical training and perform the most arduous of physical activities during their military service. They carry heavy loads on their backs and march and run long distances over varied terrain. They represent a population whose musculoskeletal system is subjected to extremely high and sustained loading [8]. According to the Atlas of Injuries in the US Armed Forces [9], musculoskeletal (orthopedic) conditions are the leading cause of disability for all three services: army (53%), navy and marine corps (63%), and air force (22%). No specific data is available for elite infantry soldiers.

We undertook a comparison study of the effect of the extremes of very high versus very low sustained musculoskeletal loading beginning at a young age on the musculoskeletal health of the lower back and knees at middle age using MRI. We compared a group of elite infantry soldiers who were under surveillance since the beginning of their military training in 1983 [8] with a group of full time Torah scholars who were deferred from military service at the same time and maintained a sedentary lifestyle since then.

## 2. Materials and Methods

Military service is universal among the male Jewish population in Israel. At the age of 17, potential recruits are called up for a preinduction assessment. Each recruit is given a health profile based on a review of his prior medical history and a detailed physical examination. The profile is given along with codes of the specific health problems on which it is based. The profile is updated on an ongoing basis during the course of compulsory military service and afterwards during the period of reserve military service eligibility. Recruits with high profiles can serve in elite infantry units if they volunteer and pass physical and psychosocial tests.

In February 1983, 295 elite male Israeli infantry recruits who were participants in a prospective study of stress fractures began their infantry basic training [8]. Recruits who subsequently completed advanced infantry training and their unit training became elite infantry soldiers.

During basic training, 31% of the recruits sustained stress fractures reflecting the difficulty of their training [8]. All were treated with 2 to 10 weeks of limited duty and then completed their training. The health profile status of these soldiers over the course of 25 years from the time of their military induction was obtained from the central Israeli Defense Forces computer. Soldiers whose health profiles changed because of back and/or knee problems during this period were identified. Whether these changes allowed them to remain elite infantry soldiers or to serve as noninfantry combat soldiers or required them to serve in noncombat positions was determined.

The health of the lower backs and knees of a group of soldiers from the 1983 study was compared to a control group of full time Torah scholars who were eligible for induction into the army in the period from 1981 to 1985 but never served in the military. These Torah scholars had army health profiles which allowed them to volunteer for elite combat units. The study was approved by the Ethics Committee of the Hadassah University Hospital and of the Israel Defense Forces Medical Corps and was registered with ClinicalTrials.gov (NCT00270608). Informed written consent was given by all study participants.

### 2.1. MRI Study Group 1 (Elite Infantry Recruits)

A list of subjects from the 1983 study group who completed three years of compulsory military service as elite infantry soldiers and who continued to serve at least once a month for the next seventeen years in the military reserves was obtained from the Israeli Defense Forces. From this list of 51 subjects, 26 were randomly selected and contacted by the study's senior author who served as their orthopedist in 1983 and were requested to participate in the study. The database from the 1983 study for these soldiers was preserved by the senior author of this study and was available for analysis.

### 2.2. MRI Study Group 2 (Ultra-Orthodox Torah Scholars)

A list of subjects from the Jerusalem region who received high health profiles, whose scheduled military induction in 1982–1985 was permanently deferred because they were Torah scholars and who continued to study Torah full time (≥12 hours per day) at least until the age of 25, was obtained from the Israeli Defense Forces. From this list of 52 subjects, 20 were randomly selected and contacted by a rabbi and asked to participate in the study.

## 3. Assessment of Current Musculoskeletal Health

Two subjects were evaluated biweekly between September 2007 and August 2008. After signing informed consent, each participant underwent the following assessments: a general questionnaire was administered orally, detailing military service, education, Torah studies, social economic status, medical history, orthopaedic injuries, and maternal and paternal origins (Ashkenazi or Sephardic). Current alcoholic consumption was assessed by recording the number of glasses of wine, beer, and hard liquor drunk weekly. The total intake was converted into units of eight ounces size with 14% alcoholic content. Smoking history was assessed by the lifetime pack years smoked. This was calculated by multiplying the number of packs of cigarettes smoked per day by the number of years the subject smoked. If the subject had changed smoking intensity, it was taken into account.

The questionnaire of Aadahl and Jørgensen [10] was orally administered to quantify the average daily physical activity of subjects. The intensity of every specific activity was scored in units of metabolic equivalents (METS).

The Israeli Army sports activity questionnaire in use since 1988 was orally administered to subjects to determine what sport activities they had participated in continuously for the previous five years and for how many hours each week. If the sports activity was not continuous over the previous five years, it was not recorded.

Height and weight were measured on a doctor's balance scale (Detecto, Webb City, MO), and the body mass index was calculated.

An MRI was taken of the spine (T10-S1) using a Siemens 3T Trio MRI (Erlangen, Germany). The results were read by a single neuroradiologist. Disk degeneration was scored based on the criteria of Pfirrmann [11] as listed in Table 1 for each level from T12 to S1. A numerical score which was one number less than the Pfirrmann grade was given for each disk level. A combined score for all levels was calculated.

An MRI of the right knee was taken using a Siemens 3T Trio MRI (Erlangen, Germany). The results were read by a single musculoskeletal radiologist blinded to the identity of the subjects. Each study was scored for degenerate changes according to the criteria of WORMS [12]. This scoring system incorporates fourteen features: articular cartilage integrity, subarticular marrow abnormality, subarticular cysts, subarticular bone attrition, marginal osteophytes, medial and lateral meniscal integrity, anterior and posterior cruciate integrity, medial and lateral ligament integrity, synovitis/effusion, intra-articular loose bodies, and periarticular cysts/bursitis. Meniscus tears were assessed by the criteria of Englund et al. [2].

## 4. Statistical Analysis

Statistical analysis was made using the Statistical Analysis System version 9.1 (SAS Institute Inc., Cary, N. C., USA). Nominal data were assessed with Chi-square. Interval variables were assessed visually for normal distribution and using the Kolmogorov-Smirnov test with a cutoff of 0.15. Normally distributed interval data were compared across the groups, using Student's *t*-test. Skewed data and ordinal data were compared using the Mann-Whitney rank sum test. The Pearson correlation coefficient was used to assess the correlation between variables studied.

## 5. Results

Of the 295 recruits from the 1983 study, three received profiles because of knee problems and thirteen because of low back problems (*P* = 0.01), during the 25-year period since beginning basic training. Two of those with knee problems became noninfantry combat soldiers and one a noncombat soldier. One soldier with a back problem remained an infantry soldier, eight became noninfantry combat soldiers, and 4 became noncombat soldiers.

Twenty-six soldiers from the 1983 induction group were contacted by phone by the study's senior author who was one of their army physicians in 1983. Twenty-five subjects agreed to participate. All of the 20 subjects contacted by the rabbi to participate in the study agreed to do so. Twenty soldiers and 17 Torah scholars had both maternal and paternal Ashkenazi origins.  Table 2 shows that the general characteristics of the two study populations were similar except for their military service histories and a higher mean weekly alcohol consumption among the soldiers group. No study subject exceeded moderate alcohol consumption.

The current anthropometric and physiological measurements (Table 3) were similar between the two populations, except that soldiers were on the average 3 cm taller (*P* = 0.02).

There was a statistically significant difference between the current mean daily energy expenditure of Torah scholars and soldiers (*P* = 0.001). The soldiers currently expended almost one-third more energy daily as expressed in metabolic equivalents than the Torah scholars (Table 4). This difference was mostly due to a greater participation in sport activities by the soldiers (*P* = 0.001).

Generally, the Torah scholars avoided participation in sport as can be seen from a compendium of their continuous sport activity for the previous five years (Table 5), considering it a waste of valuable study time. In addition to sport participation, the soldier cohort had one month of combat reserve military service yearly since finishing their regular military service.

There was no difference between the level and frequency of back or knee pain between the cohorts. Sixty-eight percent of soldiers and 70% of Torah scholars reported that they never suffered from back pain. Seventy-six percent of soldiers and 75% of the Torah scholars reported that they never suffered from knee pain. No difference in the degree of degenerative knee changes was found between the cohorts according to the WORMS score (Table 6). There was no statistical difference in the incidence of meniscus tears between the cohorts. Three soldiers had meniscus tears, 5 had a suspicion of medial meniscus tears, and one had undergone a partial menisectomy. Two Torah scholars had meniscal tears, and two had a suspicion of medial meniscal tears (*P* = 0.24). Soldiers had a significantly higher degree of T12-S1 disc degenerative changes than Torah scholars (*P* = 0.003) according to the Pfirrmann score (Table 6). One soldier and one Torah scholar had facet joint degenerative changes.

By univariate analysis, no statistically significant correlations were found between any of the anthropometic, physiological measurements or MET scores made in this study and the WORMS or Pfirrmann scores.

## 6. Discussion

In this longitudinal study, the musculoskeletal health of knees and the lower back at middle age of two groups who were at the extremes of physical lifestyles since age 18 was compared. The study shows that very high skeletal loading did result in an increase in lumbar disk degeneration as assessed by the Pfirrmann score [11] but not degenerative knee changes as assessed by the WORMS score [12]. Over the past 25 years, soldiers from the 1983 study had a significantly greater incidence of back problems, requiring changes in their health profiles than knee problems.

The extremely physically active group in this study was composed of former elite infantry soldiers who were subjects in a 1983 prospective study of stress fractures [8]. Their training was so physically demanding that 31 percent were found to have sustained stress fractures during basic training. The sedentary group in this study was composed of Torah scholars. They had the same military health profiles when they were 18 years old as the elite infantry soldiers. Instead of doing military service, they received deferment and continued to study Torah. The extent of their physical activity was usually limited to walking to their place of study and domestic activities.

During the three years of compulsory military service, the soldiers marched long distances over difficult terrain carrying heavy loads. These loads were not supposed to exceed 40% of their body weight but often exceeded 50% of their body weights. During this same period, the Torah scholars sat long hours studying. After completing three or more years of regular army service, the soldiers continued to serve a month each year in reserve infantry units until the age of forty. During this time, they trained in the field wearing ceramic vests, while carrying their packs and weapons.

The great difference in the physical activity levels between the groups continued until the time of the 25-year review done in this study. The mean daily energy expenditure of the soldiers was a third higher than that of the Torah scholars. Most of this difference was because few Torah scholars participated in any sport or exercise activities other than walking to their place of study. The former elite infantry soldiers continued to do endurance sports such a running, mountain and road biking, and basketball.

In the current study, subjects were from a very narrow and well-defined genetic pool. Most were Ashkenazi Jews, with only a small portion being Sephardic Jews. Recent genetic studies have shown that these two populations are closely genetically related [13]. Because both groups in this study also had similar body weights and smoking histories, the study is an effective model for studying the single variable of the effect of extremes in physical lifestyle on the health of the lumbar spine and knees in middle age.

In vivo intradiscal pressure measurements allow us to understand how disc stress varies with different activities. During relaxed standing, the pressure has been measured to be 0.5 megapascals. In laboratory conditions, the intra disk pressure while lifting a 20 kg weight with the back flexed has been measured to be 2.3 megapascals [14]. The intradiscal pressure while carrying loads greater than 40% of body weight and simultaneously marching or running over uneven terrain as occurred in the soldier cohort in this study is not known.

Dimitriadis et al. [15] studied the effect of a one-hour period of repetitive loading on disk height. They examined the lumbar spines of 25 long-distance runners using an upright MRI scanner before and after running for one hour. After the run, a significant reduction in disc height was found with the runners imaged seated upright and leaning forward and backward. No assessment was made of how long it took the affected discs to return to their baseline height measurement.

In a longer term study, the effect of extremely high musculoskeletal loading on lumbar discs was evaluated before and after 14 weeks of Navy SEAL training [16]. The Navy SEAL training is of similar intensity to the training of the elite infantry soldier cohort in the present study. No MRI interval changes in the lumbar spines were found over the 14-week period.

Pye et al. [17] studied the influence of weight, body mass index, and lifestyle factors on radiographic features of lumbar disc degeneration in Caucasian Scottish men and women older than 50 years of age. They found that none of these factors affected disk height, but increased subject weight was associated with an increase in lumbar osteophytes.

Disc degeneration appears to be a complex process, combining mechanical, biochemical, histological, and genetic factors [18]. Twin studies have shown that disk degeneration is primarily determined by genetic influences [3]. Despite extraordinary discordance between twin sibling in leisure time and physical loading conditions in adulthood, little effect on disc degeneration was observed. The effects of smoking on twins with large discordance in smoking exposure demonstrated a small increase in disc degeneration associated with smoking. No evidence was found to suggest that exposure to whole body vibration through motorized vehicles leads to accelerated disc degeneration.

In the present study, the two groups were at the extremes of musculoskeletal loading. The soldiers at 25-year followup appear to have paid a price in their lumbar spines for this extreme loading. The amount of degenerative changes in the lumbar spine as evaluated by the Pfirrmann score was 27% higher in the soldiers than that of the Torah scholars.

The effect of short term high loading on knees has been studied by Hohmann et al. [19]. They performed MRI studies of the knees on six recreational and two semiprofessional runners before and after a marathon run. In seven of the subjects the prerun and postrun studies did not demonstrate any marrow edema, periosteal stress reactions, or joint effusions. One runner who had undergone an anterior cruciate ligament reconstruction 18 months prior to the race demonstrated a small effusion in the reconstructed knee before and after the race.

In a longer term study, the knees of ten Navy SEAL recruits were evaluated by MRI before and after a demanding 14-week training course [16]. Prior to this course, the recruits had done a preliminary less demanding training course. Before the training course, one knee had a small lateral femoral condyle cartilage lesion. The lesion was unchanged after the training course. Nine of ten knees had prepatellar swelling, five had increased joint fluid and two bone edema in the pretraining MRI. Follow-up magnetic resonance imaging showed improvement in the prepatellar swelling in eight soldiers, no change in one soldier, increased knee effusion, and a new medial femoral condyle bone edema in another.

Englund et al. [2] randomly recruited 991 middle-aged and elderly subjects to undergo MRI of their right knee. Males in the age group from 50 to 59 were found to have a 32% prevalence of meniscus tears. The majority of these tears were asymptomatic. In the current study, the population was in their early forties. Thirty-two percent of the soldiers and 20 percent of the Torah scholars were found to have meniscus tears by the same MRI criteria as used by Englund et al. One soldier had undergone a partial menisectomy. The study size of the current study is too small to directly compare the prevalence of meniscus tears with that of the Englund et al. study [2].

There are several possible explanations for the greater long term resilience of the meniscus compared to that of the lumbar disks found in this study among the high loading group. One is that the heavy back packs carried by the soldiers in many of their activities repetitively placed higher stresses on the lumbar intervertebral disks than on the meniscus and thereby led to increased fatigue failure. Another explanation is that the meniscus may have better fatigue failure characteristics at repetitive high loading than the lumbar intervertebral disk.

## 7. Conclusions

This study compares the effect of extreme physical activities with an inactive life style on knees and the lower back at middle age using elite infantry soldier and Torah scholar cohorts. From an engineering stand point, this represents a test of very high versus very low sustained loading on these structures. The strength of the study is the uniform genetic backgrounds of the subjects. The main study limitation is the relatively small number of subjects in each of the two study cohorts. Even with this limitation, statistically significant higher lumbar disk degeneration was found in the elite infantry cohort. In spite of the extreme differences in the musculoskeletal loading between the cohorts, there was surprisingly little difference found by MRI criteria, in the amount of degenerative changes in both their knees and lumbar spines at middle age. The study indicates that the knee may possibly be more resilient to sustained heavy loading than are lumbar disks.

## Figures and Tables

**Table 1:** The Pfirrmann score for disc degeneration.

Grade	Structure	Distinction of nucleus and anulus	Signal intensity	Height of intervertebral disc
I	Homogeneous, bright white	Clear	Hyperintense, isointense to cerebrospinal fluid	Normal
II	Inhomogeneous with or without horizontal bands	Clear	Hyperintense, isointense to cerebrospinal fluid	Normal
III	Inhomogeneous, gray	Unclear	Intermediate	Normal to slightly decreased
IV	Inhomogeneous, gray to black	Lost	Intermediate to hypointense	Normal to moderately decreased
V	Inhomogeneous, black	Lost	Hypointense	Collapsed disc space

**Table 2:** Background characteristics of study populations (mean ± SD or mean, median and IQR).

Variable	Torah Scholars *N* = 20	Soldiers *N* = 25	*P* value
Age (years)	43.7, 43.0, 41.5–45.5	43.2, 43.0, 43.0–43.0	0.9
Weight (kg in 1983)		72.6 ± 5.9	
Recalled weight (kg in 1983)	73.0, 73.5, 70.0–78.0	73.7, 74.0, 68.0–77.0	0.9
Years of education	15.7, 14.0, 16.0–18.0	15.9, 16.0, 16.0–18.0	0.9
Packs years smoking	4.3, 0.2, 0.0–7.5	3.0, 0.0, 0.0–1.5	0.26
Alcohol consumption (units/wk)	0.3, 0.0, 0.0–1.0	2.1, 1.0, 0.0–2.0	0.01

**Table 3:** Current anthropometric measurements and physiological measurements (mean ± SD or mean, median and IQR).

Variable	Torah Scholars *N* = 20	Soldiers *N* = 25	*P* value
Height (cm)	176, 1.76 172.7–178.0	179.2, 179.0 176.5–182	0.02
Weight (kg)	85.9, 85.3 74.1–91.3	84.5, 85.0 78.0–90.0	0.7
BMI	27.8, 27.2 23.8–29.5	26.3, 26.6 25.0–28.0	0.3

**Table 4:** Current 24-hour energy expenditure in metabolic equivalents (METS) of Torah scholars versus soldiers (mean, median, and IQR).

Variable	Torah Scholars *N* = 20	Soldiers *N* = 25	*P* value
Mean daily total energy expenditure (METS)	33.6, 33.4 32.8–35.1	44.1, 41.8 37.5–49.3	<0.001
Mean daily energy expenditure in sports (METS)	1.9, 1.2 0–3.3	8.1, 7.9 4.0–11.0	<0.001

**Table 5:** Compendium of continuous sports participation for the previous five years of Torah scholars versus soldiers.

Sport Activity	Torah Scholars		Soldiers	
	*N* = 20		*N* = 25	
	No. of Participants	Hours/Week	No. of Participants	Hours/Week
Running (>5 Km runs)	0	0	13	4.6
Running (≤5 Km runs)	0	0	2	1.5
Road or mountain biking	0	0	12	2.6
Swimming	1	4	8	1.9
Basketball	0	0	6	2.8
Calisthenics/weight lifting	2	3.3	4	1.7
Tennis/sQUASH	0	0	2	0.8
Wind surfing	0	0	1	4

**Table 6:** Knee and Back complaints and knee and back MRI findings (mean ± SD or mean, median and IQR).

Variable	Torah scholars (*N* = 20)	Soldiers (*N* = 25)	*P* value
Knee pain score (1–4)	0.5, 0, 0.0–0.5	0.6, 0, 0.0–0.0	1.0
Back pain score (1–4)	0.8, 0, 0.0–2.0	0.7, 0, 0.0–1.0	1.0
Worms score	2.0, 1.5, 0.0–3.5	1.8, 1.0, 0.5–2.7	0.7
Pfirrmann score	6.7, 7.0, 5.0–7.0	8.6, 9.0, 7.0–10.0	0.003
